# Understanding family planning outcomes in northwestern Nigeria: analysis and modeling of social and behavior change factors

**DOI:** 10.1186/s12889-021-11211-y

**Published:** 2021-06-17

**Authors:** Paul L. Hutchinson, Udochisom Anaba, Dele Abegunde, Mathew Okoh, Paul C. Hewett, Emily White Johansson

**Affiliations:** 1 Department of Global Community Health and Behavioral Sciences, School of Public Health and Tropical Medicine, Tulane University, New Orleans, Louisiana 70112 USA; 2 Population Council, Washington, DC USA; 3 Johns Hopkins Center for Communication Programs, Baltimore, MD USA

**Keywords:** Family planning, Nigeria, Social and behavior change

## Abstract

**Background:**

Northwestern Nigeria faces a situation of high fertility and low contraceptive use, driven in large part by high-fertility norms, pro-natal cultural and religious beliefs, misconceptions about contraceptive methods, and gender inequalities. Social and behavior change (SBC) programs often try to shift drivers of high fertility through multiple channels including mass and social media, as well as community-level group, and interpersonal activities. This study seeks to assist SBC programs to better tailor their efforts by assessing the effects of intermediate determinants of contraceptive use/uptake and by demonstrating their potential impacts on contraceptive use, interpersonal communication with partners, and contraceptive approval.

**Methods:**

Data for this study come from a cross-sectional household survey, conducted in the states of Kebbi, Sokoto and Zamfara in northwestern Nigeria in September 2019, involving 3000 women aged 15 to 49 years with a child under 2 years. Using an ideational framework of behavior that highlights psychosocial influences, mixed effects logistic regression analyses assess associations between ideational factors and family planning outcomes, and post-estimation simulations with regression coefficients model the magnitude of effects for these intermediate determinants.

**Results:**

Knowledge, approval of family planning, and social influences, particularly from husbands, were all associated with improved family planning outcomes. Approval of family planning was critical – women who personally approve of family planning were nearly three times more likely to be currently using modern contraception and nearly six times more likely to intend to start use in the next 6 m. Husband’s influence was also critical. Women who had ever talked about family planning with their husbands were three times more likely both to be currently using modern contraception and to intend to start in the next 6 m.

**Conclusion:**

SBC programs interested in improving family planning outcomes could potentially achieve large gains in contraceptive use—even without large-scale changes in socio-economic and health services factors—by designing and implementing effective SBC interventions that improve knowledge, encourage spousal/partner communication, and work towards increasing personal approval of family planning. Uncertainty about the time-order of influencers and outcomes however precludes inferences about the existence of causal relationships and the potential for impact from interventions.

## Background

Nigeria currently has one of the highest fertility rates in the world [1], with the northwest region experiencing the highest rates within the country [2]. The 2018 Nigeria Demographic and Health Survey (NDHS) indicated that the total fertility rate in the northwest of the country was 6.6 live births per woman, and that women aged 40 to 49 years averaged 8.3 births in their reproductive lifetimes [2]. This high-fertility situation places women at greater risk of birth complications and maternal mortality. Nigeria currently has more maternal deaths annually than any other country in the world [3] and the fourth highest maternal mortality ratio [4].

Contraceptive use to limit or space births is not the norm in this region. In the 2018 NDHS, only 6.2% of married women in the northwest were currently using any form of modern contraception, and the majority of married women - 68.7% - reported no need for family planning for either spacing or limiting [2]. Much of this absence of demand for family planning can be attributed to social norms for high fertility, pro-natal cultural and religious beliefs, misconceptions about contraceptive methods, and gender inequalities.

### Role of high parity norms

In this region, the desire for large families is extensive, reflected in a mean ideal number of children of 7.5 [2]. This is nearly three children more than the ideal in the south of the country [5]. Even among high parity couples, the desire to continue having children prevails. According to the 2018 NDHS, 61.6% of women with six or more children in this region wanted more children. Among men with six or more children, that percentage was even higher; 89.1% desired more [2].

Social norms driving high fertility in the northwest are tied in part to perceptions of its social advantages, such as signaling greater wealth and status, ensuring the survival of family names, and broadening social networks and influence. Large family size is believed to both represent and engender wealth, influence, respect, and fame [6]. Further, large families are perceived to have economic benefits, such as serving as social insurance for parents as they age and contributing household labor or income from market-based employment [6]. Son preference may further drive high fertility [7].

### Role of religion

In the north, where the majority of the population is Muslim, religious beliefs drive high fertility [5–8]. Izugbara and Ezeh (2010) note that many women believe that high fertility honors Allah. Specifically, one way “to serve God with fertility is to give birth to several children who will worship Him and secure the future of Islam” [6]. Similarly, Obasohan [9] highlights the cultural belief that God places children in the womb and “until they are given birth to, you do not stop.”

### Role of contraceptive myths

Further affecting high fertility rates in northwestern Nigeria are misconceptions and negative perceptions about family planning use, such as beliefs that contraceptives are dangerous to a woman’s health [10–12], that they can harm a woman’s womb [10, 13, 14], that they can inhibit subsequent fertility [10, 12] or that they can cause cancer [6].

### Role of gender inequalities

Fertility in northwestern Nigeria is also driven by gender power imbalances, fostered by patriarchal social structures in which women have limited autonomy over most decisions, including those affecting marriage, health and fertility [7, 15]. Men are often the final decision-makers on important household matters, including those related to “household purchases, health of family members, timing of pregnancies, family size, and education of children” [16]. As the decision-makers on family size, men ultimately determine contraceptive use through their fertility desires and approval or disapproval of contraception [7, 17].

Exacerbating power differentials are the low levels of female education and patterns of early marriage. In the northwest, nearly two thirds of adult women have no formal education, and only 29% are considered literate [2]. Forced and early child marriage are common [18], and many girls are married as young as 12. The median age at first marriage is approximately 15.9 years. The median age for men, in contrast, is 25.3 years, revealing considerable age differences, and hence power differentials [2]. In this context, women are valued largely for their reproductive functions [7, 18].

From a woman’s perspective, “fertility is one mechanism by which women can impart some control over marital situations that are largely beyond their control” [6]. High parity is perceived as a mechanism to ensure marital stability, and protection and financial support from their spouse [9, 18]. Wives often see having many children as a way to discourage husbands from taking on other wives [6], which can affect a wife’s standing within the polygynous familial structure [19]. In polygynous marriages, resources and wealth are generally distributed to wives based on the number of children they have, both on a daily basis and at the husband’s death, thereby limiting incentives to use contraception [6]. Researchers have identified conjugal relationship dynamics as explaining 11% of the variation in contraceptive use between northern Nigerian states and southern Nigerian states [20]. Further, low fertility can have dire consequences for women as husbands “may cite limited childbearing as an excuse to marry additional women and to divorce their existing wives” [6].

### Family planning demand

In northwestern Nigeria, decisions about contraceptive use are inextricably linked to this complex interaction of high fertility desires, social norms, and contraceptive myths, as well as economic factors such as financial security, income streams, and the costs of health services [13, 20–22].

This work examines several family planning outcomes and their relationships with theorized determinants of contraceptive use. It builds upon the Ideational Theory of Behavior Change [23–26], which in turn builds upon other behavior change theories, including the diffusion of innovations [27], the theory of planned behavior [28], social cognitive theory [29, 30], and the transtheoretical model [31]. These behavioral models highlight the roles of multiple direct and indirect influencers of behaviors, including intentions, environmental constraints, skills, attitudes, norms, identity, emotion and self-efficacy, with the first three factors believed to be necessary and sufficient for a behavior to occur while the latter five factors influence the strength and direction of intentions [32].

This study focuses in particular on several key components of these theories that may be of particular relevance for the design and implementation of behavior change programs in northwestern Nigeria that seek to influence contraceptive use, including interpersonal discussions between couples, approval of family planning, and contraceptive knowledge.

### Interpersonal communication among couples

We focus on the role of communication among couples about family planning because of its established association with a greater likelihood of contraceptive use in certain contexts [13, 22, 33]. Nonetheless, contraceptive discussions are not the norm in this region [19], and discussions about family planning with young or unmarried persons are often considered inappropriate (Adebayo et al., 2011). The Nigerian Urban Reproductive Health Initiative (NURHI) reported that less than a third of married women in northern Nigeria discussed family planning with spouses at least once within the past six months [14].

While husbands influence fertility decisions, most issues of reproductive health are considered a woman’s domain [7, 17]. Hence, a woman is expected to be the one to initiate conversations about family planning [17, 34], even though these conversations come with risk for her. Trepidation about discussing family planning inhibits many couples from discussing family planning and introducing the topic with a husband ([7].

### Approval of family planning

We focus as well on approval of contraception – or its absence – as a facilitator of contraceptive use, as shown in previous studies [35, 36]. In northern Nigeria, strong cultural and religious forces limit the acceptability of modern contraception among large swaths of the population. A 2003 study of married men in northern Nigeria found that nearly two thirds of men disapproved of the concept of contraception [37], a finding mirrored by others [8].

While many studies have looked at the role of contraceptive approval in affecting contraceptive decisions, particularly by partners [36, 38], few studies have looked specifically at the determinants of approval itself. Because contraceptive use must fit within a person’s values, approval is a necessary (but not sufficient) condition for use. Its examination in the context of decisions about contraceptive use is therefore critical. Stages of change theories, such as the transtheoretical model, consistently highlight the process of developing a positive attitude toward an intended behavior as a prerequisite to engaging in the behavior [24, 31, 39]. For actions with significant negative associations, behavior change programs necessarily must work to improve attitudes towards the behavior. Achieving improved acceptance of contraception remains an important intermediate goal of those programs.

### Contraceptive intentions

We also focus on contraceptive intentions as an outcome because of the strong role that they play in major behavioral theories, although measurement of intentions often conflates the time order between intentions and contraceptive use. As with approval, we treat intentions as a necessary but not sufficient condition for contraceptive use; women are unlikely to inadvertently begin using contraception and hence intent is a necessary condition. Understanding the factors associated with this necessary step are critical for understanding contraceptive uptake.

Northern Nigeria has persistently low contraceptive intentions because the majority of fertility-aged women desire to become pregnant [2, 40]. Even though intentions to use are low, previous analyses have shown that they are malleable and can be influenced by greater self-efficacy, reductions in contraceptive myths, and social influences [26]. In other contexts, intentions to use postpartum family planning (PPFP) have been shown to be associated with past use, acceptability of use, and of partner acceptability of contraception [41].

### Objective

This paper contributes to the extant literature on contraceptive use in a high-fertility environment by quantifying the importance of the myriad factors highlighted in behavior change theories, not just on contraceptive use but also on intermediate determinants of contraceptive use, including contraceptive intentions, interpersonal communication, social influences, and approval. This paper recognizes the importance of these intermediate determinants in previous reviews of contraceptive use in Nigeria but notes that they have seldom been studied as outcomes themselves, a key aim of this paper. Further extending previous analyses, this paper models how social and behavior change programs may effectively change contraceptive behaviors by targeting these myriad influences.

## Methods

### Data

Data for this study were collected as part of a baseline survey conducted by the USAID-funded Breakthrough RESEARCH project (B-R) as part of a three-year evaluation of the Breakthrough ACTION / Nigeria (B-A/N) project. From 2019 to 2022, Breakthrough ACTION/Nigeria, which is also funded by USAID, will operate in 11 states of Nigeria and the Federated Capital Territory (FCT) of Abuja. B-A/N is an integrated social and behavior change program targeting family planning, malaria and maternal, newborn, and child health and nutrition. The B-A/N program has three core components: 1) advocacy outreach to opinion leaders and community influencers at State and Local Government Area (LGA) levels; 2) direct engagement of community members through household visits and community dialogues directed at target populations, with referrals for services as needed; and 3) complementary integrated SBC messaging through mass, mid-media and mobile phones.

Data were collected through face-to-face household interviews with women aged 15 to 49 years with a child under 2 years in the northwestern states of Kebbi, Sokoto and Zamfara in September 2019, prior to B-A/N program implementation. The data are representative of populations within B-A/N programming areas, but not across the states at large.

### Sampling

We conducted a two-stage cluster-sample, cross-sectional survey of women with a child under 2 years. The sampling frame for the study was developed from areas to be served by interventions of the Breakthrough Action project over the period 2019–2022. Because this survey was intended as a baseline, no Breakthrough Action activities had commenced, and hence no attempt is made here to link program interventions to health behaviors. The Breakthrough Action areas consisted of 203 wards across the states of Kebbi, Zamfara and Sokoto.

Sample sizes for the study were determined with the intent to assess differences in key outcomes across three study arms [42]. These study arms were developed as part of a larger and separate evaluation of the effectiveness of integrated versus vertical programming that divided B-A/N areas into three types: (1) Integrated high package of B-A/N interventions (e.g., larger number of household visits by community health workers and more intensive content matter across priority health areas), (2) integrated standard package of B-A/N interventions (e.g., one household visit, standard content across priority health areas) and (3) SBC malaria-only programming [42]. Study arms 1 and 2 were located in Kebbi and Sokoto states, while study arm 3 was located in Zamfara state, which was slated to receive malaria only programming from B-A/N.

To determine the required sample size and number of clusters, the Stata 16.0 sample size routine for cluster sampling (*clustersampsi*) was used [43]. The parameters specified for the sample size estimation included a power criterion of 0.80, alpha coefficient of 0.05, and intra-cluster correlations that varied by study outcomes. The key study outcomes for the calculations included prevalence of facility delivery, four or more antenatal care visits during pregnancy, measles vaccination, and pregnant women sleeping under a mosquito net. For sample size calculations, estimates of the prevalence and intracluster correlations for these outcomes were derived from the 2018 NDHS. The sample-size and cluster calculations suggested that data be collected in 108 clusters (36 per study arm), covering approximately 3000 women (1000 per arm) with a child below age two.

At the first sampling stage, a total of 108 wards across the three states were selected from among the 203 wards. Because the most recent population census in Nigeria was conducted in 2006, digital maps, produced from the Geopode database in 2019, were used to select the 108 clusters based on a geo-referenced, gridded population layer, settlement features extracted from recent satellite imagery, and neighborhood classifications based on building morphology, orientation, and density. Each cluster consisted of approximately 175 households. Clusters were mapped and listed using a community screening tool that identified households with a woman with a child below age two.

At the second sampling stage, 28 women with a child below age two who had been identified in the household listing were selected for a subsequent interview using a random number generator phone app.

### Data collection and questionnaires

Randomly selected eligible women were asked to respond to an interviewer-directed questionnaire. Fieldwork was conducted in September 2019 over a 4-week period prior to B-A/N implementation. Interviewer training occurred one week period to data collection. This training reviewed the study objectives, protocol and instruments, fieldwork procedures and ethical considerations. All interviewers participated in a questionnaire pilot exercise that tested skip patterns, checked questionnaire translation (Hausa), and assessed question appropriateness and sequence.

There were two questionnaires administered to survey participants. The household questionnaire collected information on usual resident household members and household assets and characteristics. The female questionnaire asked respondents about their demographics, reproductive history, contraceptive use, media exposure, gender norms, and ideations related to family planning. Interviews were conducted in Hausa, the predominant local language. The overall response rate among women with a child under two years was 99%.

### Variables

#### Outcomes

Several family planning-related outcomes are the focus of our study. We identified women as current users of modern contraception if they reported that they were currently using an intrauterine device (IUD), injectables, implants, pill, male condom, female condom, lactational amenorrhea method (LAM), spermicide, diaphragm, or emergency contraception or if they reported having been sterilized or that their husband had received a vasectomy. In addition to examining current use of modern contraception, we look at several family planning intermediate outcomes such as intentions to use contraception in the next six months, discussions with a partner about contraception and the number of children to have, and approval of family planning. Each of these are treated as binary variables. Intentions were measured as positive responses to the question, “Do you intend to begin using a contraceptive method in the next six months?“ These questions were only asked of current non-users of family planning. Approval of family planning was measured as a positive response to the question, “Do you personally approve of using contraception for spacing births?” No similar question was asked about approval of family planning for limiting births or use of family planning more generally. Contraceptive discussions were measured as positive responses to the question, “Have you ever talked with your husband/partner about using modern contraception?” and “Have you ever talked with your husband/partner about the number of children to have?”

#### Explanatory variables

The SBC interventions of the Breakthrough Action / Nigeria project are guided by the Ideational Theory of Behavior Change [24–26], which amalgamates the components of multiple behavior change theories and traces the effects of social and behavior change interventions (e.g., mass media, social media, interpersonal communication) through a set of core psychosocial influences that affect contraceptive behaviors and intentions. The ideational theory groups factors into three domains: cognitive (knowledge, beliefs, values, perceived risks and norms), emotional (self-efficacy) and social influences. We use this theory as a guide to variable selection for our behavioral models, as depicted in Table 1.

**Table 1:** Ideational Variables

Dimension	Domain	Likert-scale statement or question	Definition (from Kincaid et al)
Cognitive	Knowledge	- Contraception benefit for children? - Contraception benefits for self? - Side effects from using contraception are normal and usually go away in a few months. - A woman’s body is not ready for childbirth until she is 18. - Women over 35 have a higher risk of complications during pregnancy and shortly after birth.	
	Beliefs (attitude)	- Couples who use a modern contraception have better quality of life.	Beliefs about an object or behavior
	Values (attitude)	- Do you personally approve of using contraception for spacing births? - It is important that husbands and wives discuss contraception.	Values that specify its positive or negative consequences
	Contraceptive Myths	- Use of some contraceptives can make a woman permanently infertile. - Contraceptives can harm a woman’s womb. - Contraceptives can reduce a man’s sexual urge. - Contraceptives reduce a woman’s sexual urge. - Contraceptives can cause cancer. - Contraceptives can give you deformed babies. - Women who use contraception end up with health problems. - Women who use contraceptives may become promiscuous.	-
	Subjective Norms	- People will call you bad names if they know you use contraception. - q326 Religious leaders should speak publicly about modern contraception. - Most couples in my community use modern methods for spacing.	What an individual thinks others expect him/her to do as well as what an individual thinks other people are doing (social norms)
Emotional	Self-efficacy	- How confident are you to convince your husband/partner to use modern FP? - How confident are you to use a modern method even if your partner disagrees?	Beliefs in one’s capability to organize and execute the course of action required to manage prospective situations.
Social	Social influence	- Who decides if you use a contraceptive method? (self alone, partner, both) - Besides yourself, who else influences your decision to use family planning? (husband, mother-in-law, mother, health care provider)	Encompasses all interpersonal processes by which other people persuade someone to behave a certain way, as well as influence that occurs by social modeling by others

Contraceptive knowledge was measured as a woman’s identification of the benefits of contraceptive use for children and for the woman herself, such as better growth, nutrition and overall health for children and giving a woman “a chance to rest after childbirth.” We also included in our knowledge measure agreement with statements such as “Side effects from using contraception are normal and usually go away in a few months,” “A woman’s body is not ready for childbirth until she is 18,” and “Women over 35 have a higher risk of complications during pregnancy and shortly after birth.” An index of knowledge was created from the score of the first principal component using the *polychoricpca* command in Stata [44]. The sample was then divided into halves as those who had knowledge scores above and below the median. In a similar manner, we constructed an index of incorrect knowledge and belief in common contraceptive myths based on agreement with statements such as “contraceptives can cause cancer” and “women who use contraceptives may become promiscuous,” and again grouped women into two halves of low and high levels of belief in contraceptive myths.^1^

We included one measure of. contraceptive beliefs – agreement with the statement that “Couples who use a modern contraception have better quality of life” - and two measures of values – agreement with the statements that “it is important that husbands and wives discuss contraception” and “do you personally approve of using contraception for spacing births?” As discussed previously, we also examined approval as an outcome but included it in the contraceptive use, intentions and discussions models to assess how approval as an intermediate factor influences these other outcomes.

Two norms variables were included in our models, including one injunctive norm – agreement with the statement “Religious leaders should speak publicly about modern contraception.” and one descriptive norm – agreement with the statement that “Most couples in my community use modern methods for spacing.”

An important objective of the analysis was to assess a woman’s level of autonomy to make decisions about her own fertility and contraceptive use. We included two measures of social influence. First, we examined the family planning decision-making process as responses to the question, “Who decides if you use a contraceptive method? Is it mainly your decision, mainly your partner’s decision or do you both decide together?” Binary variables were created for “mainly the respondent’s decision” and “joint decision-making” relative to decision-making by the husband/partner. Secondly, we included information on specific influencers based on responses to the question, “Who else influences your decision to use family planning?” For this, we included dummy variables for husband/partner, mother, mother-in-law and health provider because these were the responses most commonly cited by women. We also included one measure of self-efficacy—self-reported confidence that a woman can use modern contraception.

Additional variables included both respondent’s and husband’s education, parity, maternal age, and whether or not a woman was currently breastfeeding. Husband’s education was reported by the women themselves. To test for the potential effects of the polygynous family structure, we included variables for whether or not a husband has other wives, categorized as only one wife, one other wife or three or more wives.

We measured wealth using an asset-based measure constructed from ownership of key consumer durables and then compiled into an index using principal components analysis [45]. Households were then categorized into quintiles from poorest to wealthiest.

### Analysis

In multivariate analyses, we specified mixed-effects logistic regression models for binary outcomes in which we model separately the log odds of each of our five outcomes as a linear combination of model covariates that include family planning beliefs, knowledge, values, perceived risks, norms, social influences and self-efficacy and a set of sociodemographic control variables [46]. As noted above, our five binary outcomes were use of modern contraception, intention to use modern contraception, discussions with husbands about fertility goals, discussions with husbands about use of family planning, and approval of family planning for birth spacing. We estimate our model using the *xtlogit, re* command in Stata 16. We include cluster-level random effects to address intracluster correlation at the ward level, which is our first stage sampling unit.

In post-estimation analysis, we use the estimated model coefficients to calculate the adjusted probability of an outcome for each respondent at given values of covariates using the *margins* command in Stata 16. For example in our modern contraceptive use regression, we predict the probability of modern contraceptive use for a woman approves of contraception for spacing and for a woman who does not, controlling for other model covariates. We also model average marginal effects at actual values of each model’s covariates using Stata’s *margins, dydx()* command. These allow us to show the effects of changes in each model’s covariates, including scenarios that are likely to be the targets of SBC programs, e.g., what would contraceptive prevalence be if everyone understood the health benefits of contraceptive use and held no contraceptive myths, if everyone approved of family planning, or if everyone felt confident to be able to use family planning. Although these are hypothetical scenarios, they allow us to show the potential – and limitations – of SBC programs that target the drivers of these family planning outcomes.

## Results

### Sample

The sample included 3000 women with a child born within two years of the survey interview (Table 2). The majority of these women, 73.9%, reported having no education. The majority of husbands, 58.3%, also reported no education but more than twice as many husbands as women had a secondary higher level of education – 25.0% versus 10.6%. Median parity was 3 children although 14.3% of women had 7 or more children. Because the sample consists of women with a child under the age of two years, the sample skews to younger aged women, and nearly all (93.0%) were currently breastfeeding.

**Table 2:** Sample Characteristics of women with a child born in the previous two years

Variable	% Freq.	***N***
**Maternal education (highest level attended)**		
None	73.9	2209
Primary	4.8	153
Secondary or higher	10.6	330
Islamic	10.7	308
Total	100.0	3000
N	3000	–
**Maternal age (in years)**		
15–24 years	40.6	1231
25–34 years	45.4	1365
35–49 years	14.0	404
Total	100.0	3000
N	3000	–
**Husband’s Education (highest level attended)**		
None	58.3	1704
Primary	6.1	192
Secondary	12.4	377
Tertiary	12.6	366
Islamic	10.7	310
Total	100.0	2949
*N*	2949	–
**Household wealth index**		
First (Poorest)	20.9	715
Second	20.3	597
Third	20.1	582
Fourth	19.0	489
Fifth (least Poor)	19.6	617
Total	100.00	3000
N	3000	–
**Parity**		
None	1.3	21
1	19.3	612
2	17.5	562
3	15.7	499
4	13.0	377
5	11.7	328
6	7.3	202
7+	14.3	399
Total	100.0	3000
Median No. of Children	3	3000
Currently breastfeeding?		
No	7.0	182
Yes	93.0	2416
Total	100.0	2598
N	2598	

Of the 3000 women, 13.3% (*N* = 393) were currently using modern contraception while an additional 14.7% (*N* = 333) intended to begin using in the next six months (Table 3). Discussions with husbands about fertility and contraceptive use were rare. Only 7.4% had ever discussed with their husbands the number of children to have while less than a quarter, 22.5%, had ever discussed contraception. Only 43% of women reported that they approved of family planning for spacing births.

**Table 3:**
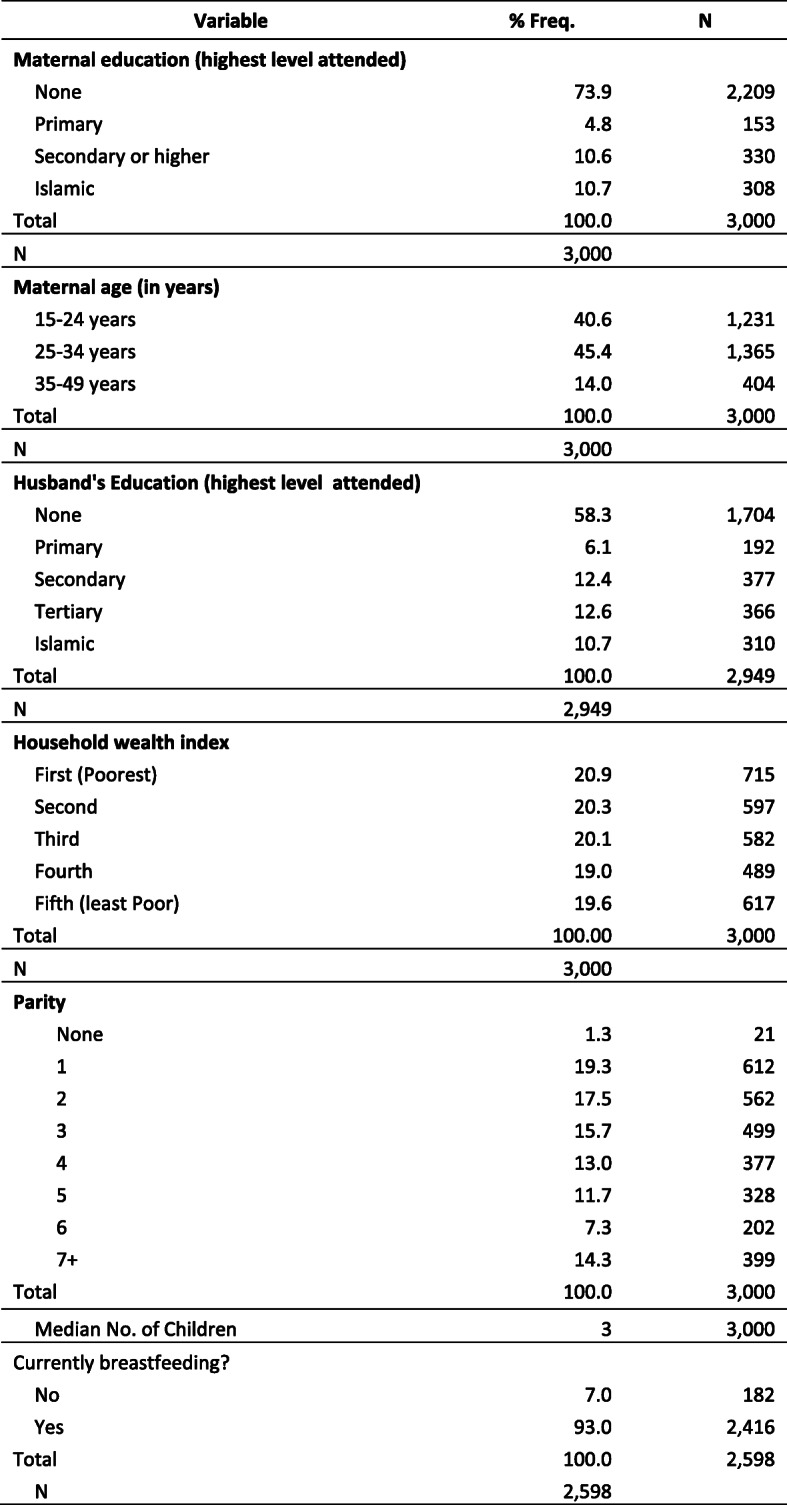
Family Planning Outcomes and Ideational Factors

Knowledge of specific benefits of contraception varied by benefit. Only 12.0% of women said that contraception provided no benefits for children, but nearly a third said that it promoted better overall health for the child and allowed for more attention by the mother. Better education and more opportunities for the child were cited by only one in 15 women. Similarly, only 11.7% of women cited no benefits of contraception for the woman herself, while approximately two-thirds reported that contraception allows a woman to get rest after a birth. Only 15.5% of women noted that contraception reduces unwanted pregnancies. Only four out of 10 women acknowledged that women over the age of 35 are at higher risk of pregnancy complications, and slightly more than one quarter of women agree that a woman’s body is not ready for childbirth until she is 18.

Contraceptive myths appear to be held by a large number of women. Nearly half of women believe that contraception can leave a woman permanently infertile, can harm a woman’s womb, can reduce both a man’s and a woman’s sexual urge, can cause cancer, can cause deformed babies, can cause health problems, and can lead a woman to become promiscuous.

Women were asked about their attitudes towards family planning. Nearly 6 out of 10 women believed that couples who use family planning have a better quality of life, and almost half believed that most couples in their community use modern contraception for spacing births. Approximately half of women believe that religious leaders should speak publicly about family planning.

There was modest evidence for women’s autonomy in decision-making. Nearly 60% of women strongly agreed that a woman should play a role in household decision-making, and nearly 70% agree that it is important that couples discuss contraception. That being said, less than a quarter of women said that decisions about family planning were solely theirs, which was a higher percentage than those who said that the decision was mainly their partners (21.7%). The majority, 54.2%, said that such decisions are made jointly with their partner. When it comes to major household purchases, the majority, 58.4%, said that such decisions were solely the husband’s.

Self-efficacy to use contraception was low. Only 37.5% of women said that they were confident that they could use modern contraception even if their husband disapproved, even though nearly half, 49.1%, felt confident that they could convince their husbands about using contraception.

In terms of influencers, 30.7% of women reported that their partner influences the contraceptive use decision, while only 3.4 and 4.7% reported that their mother-in-law and their mother respectively influenced the decision. A small percentage, 3.8%, said that they were influenced by a health provider.

The majority of modern contraceptive users (77.2%) said that the reason for using a method was that they wanted to space their births (Table 4). Only 10.2% were using contraception for limiting the number of births. The most common reasons why women were not using family planning were that fertility outcomes are “Up to God,” that there is opposition to family planning, either by the husband (21.1%) or the respondent (17.9%), or that the woman was currently breastfeeding (23.1%). Distance and cost were not cited as important barriers.

**Table 4:** Reasons for Use and Non-Use of Contraception

	Pct.	[95% Conf	Interval]	N
**Reasons for Using Contraception**				
- Prefer to wait before having another child	77.2%	66.1%	88.3%	429
- Partner wants to use contraception	32.4%	20.8%	43.9%	429
- Does not want more children	10.2%	5.0%	15.4%	429
- Health providers says shoud use	6.7%	3.2%	10.3%	429
- Protect against STIs	1.0%	−0.1%	2.1%	429
**Reasons for Not Using Contraception**				
- Up to God	24.7%	18.7%	30.7%	2571
- Breastfeeding	23.1%	16.2%	30.0%	2571
- Husband opposes	21.1%	15.9%	26.3%	2571
- Respondent opposes	17.9%	12.6%	23.2%	2571
- Wants more children	13.4%	8.5%	18.3%	2571
- Fear of infertility	8.0%	5.1%	11.0%	2571
- Interferes with body	5.9%	3.2%	8.7%	2571
- Health concerns/Fear of side effects	2.9%	1.5%	4.3%	2571
- Costs too much	0.5%	0.2%	0.8%	2571
- Difficult to get transport	0.4%	0.1%	0.8%	2571
- Distance to health facility	0.4%	0.0%	0.8%	2571

#### Mixed effects logistic regression models

Regression analysis supported previous studies indicating that ideational factors – across cognitive, emotional and social ideational domains – are associated with better family planning outcomes in northwestern Nigeria (Tables 5–9). Several factors – knowledge, contraceptive discussions with husband, and approval of family planning, showed the strongest associations across all of the outcomes. The presentation of results below focuses on the adjusted predicted probabilities in the tables. Only results that are statistically significant at better than the 5% level are discussed below.

**Table 5:**
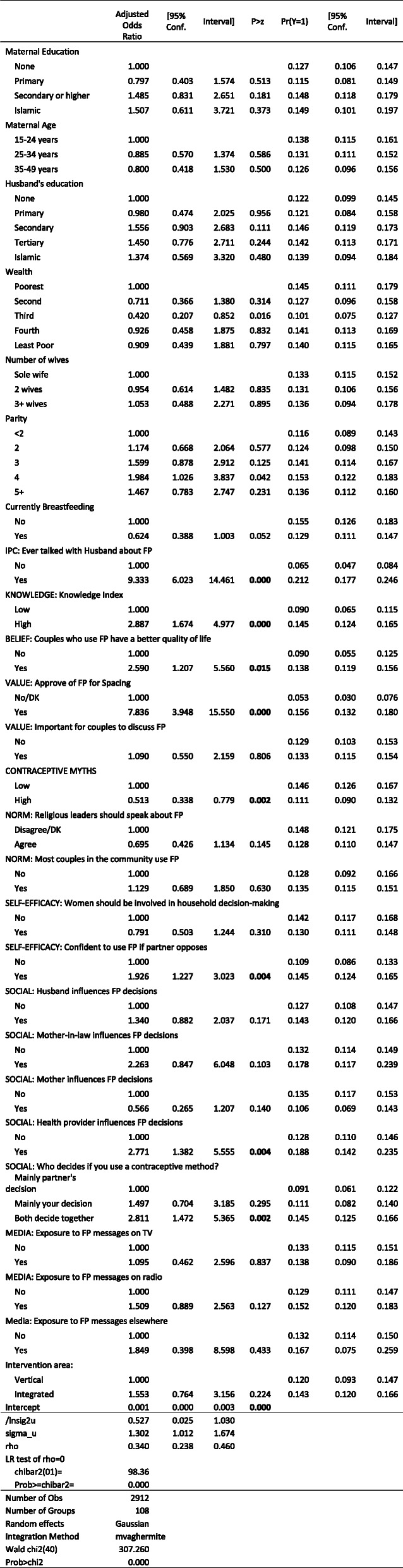
Mixed-Effects Logistic Regression: Current Modern Contraceptive Use

#### Modern contraception

The use of modern contraception appeared to be driven largely by cognitive factors – knowledge, approval, and beliefs (Table 5). Women who had greater family planning knowledge were more likely than those with poor family planning knowledge to be using modern contraception (14.5% versus 9.0%), while women holding contraceptive myths were only about three-quarters as likely to be using modern contraception as those not holding such myths (11.1% versus 14.6%) . Women who approved of family planning for spacing were three times (15.6% versus 5.3%) more likely to be using modern contraception than those who did not approve. Women who believe that couples who use family planning have a better quality of life were nearly five percentage points more likely to be using modern contraception (13.8% versus 9.0%) than those who do not hold that belief. Women with greater self-efficacy to use contraception were 3.6 percentage points (14.5% versus 10.9%) more likely to be using modern contraception, although it is not possible to determine whether self-efficacy helped drive contraceptive use or self-efficacy was developed through the process of using modern contraception. While only 3.8% of women were influenced by health providers, these women were six percentage points (18.8% versus 12.8%) more likely to be using modern contraception. Norms – as measured by the variables in our model - did not appear to be associated with contraceptive use.

#### Contraceptive intentions

The factors driving intentions to use modern contraception largely mirror those for current use (Table 6). Women who approved of family planning were nearly six times (20.6% versus 3.5%) more likely to intend to start using contraception in the next 6 months, while women believing contraceptive myths were only two-thirds as likely as women who did not hold contraceptive myths (10.5%% versus 15.8%). Confidence to use family planning helped as well; women who expressed confidence were 5.1 percentage points (15.9% versus 10.8%) more likely to intend to start. Unlike for current contraceptive use, the effects of social influences on contraceptive intentions, however, appeared to be negligible.

**Table 6:**
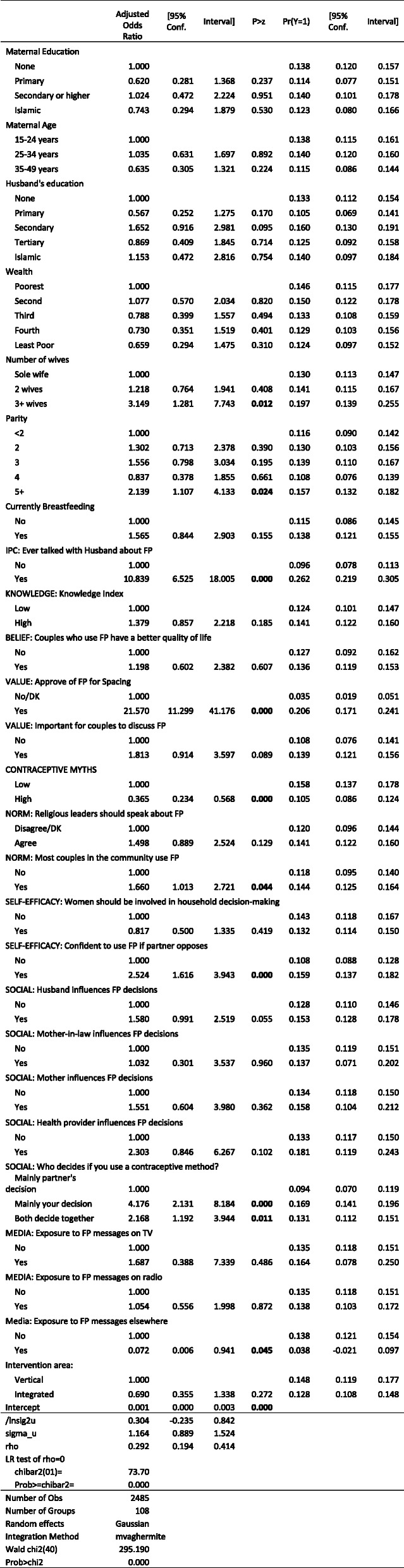
Mixed-Effects Logistic Regression: Intention to Use Modern Contraception in the next 6 months

#### Interpersonal communication with husbands

The likelihood of discussions with husbands – both about the number of children to have and use of family planning – was associated with factors across the entire ideational spectrum (Tables 7 and 8). Again, knowledge and approval were important. Women who approved of family planning were nearly three times more likely to discuss children (10.0% versus 3.8%) and family planning (28.8% versus 11.0%). Women with high knowledge were 3.5 percentage points more likely to discuss children (8.8% versus 5.3%) and 6.2 percentage points (24.0% versus 17.8%) more likely to discuss family planning. Self-efficacy to use family planning was also associated with both outcomes. The influence of mothers – but not mothers-in-law – was also observed for both outcomes. Health provider influence was associated with a 5.3 percentage point (27.2% versus 21.9%) greater likelihood of discussing family planning with one’s husband.

**Table 7:**
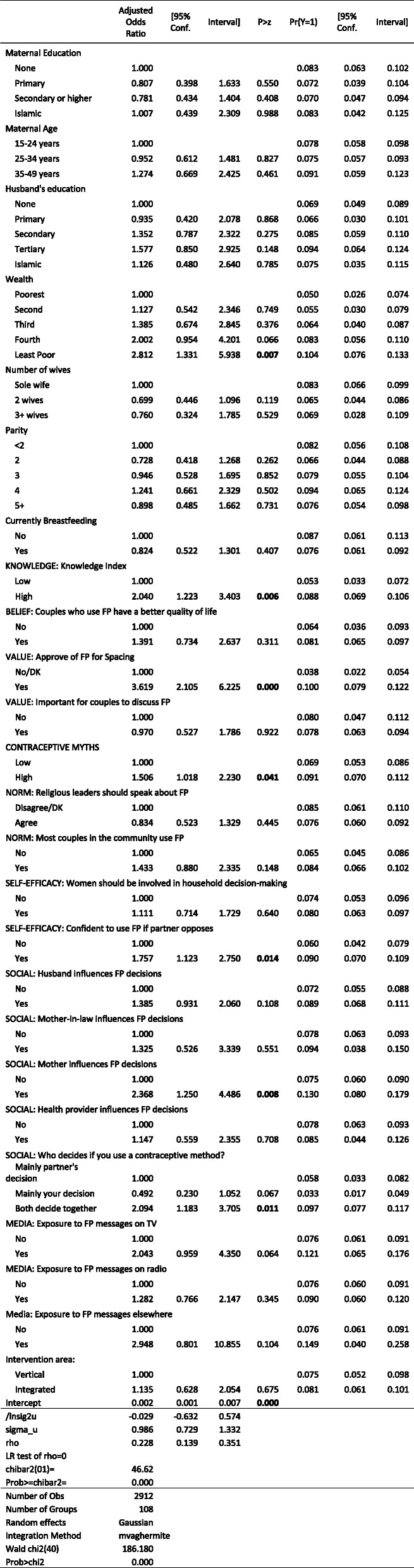
Mixed-Effects Regression: Ever spoken with husband about the number of children to have

**Table 8:**
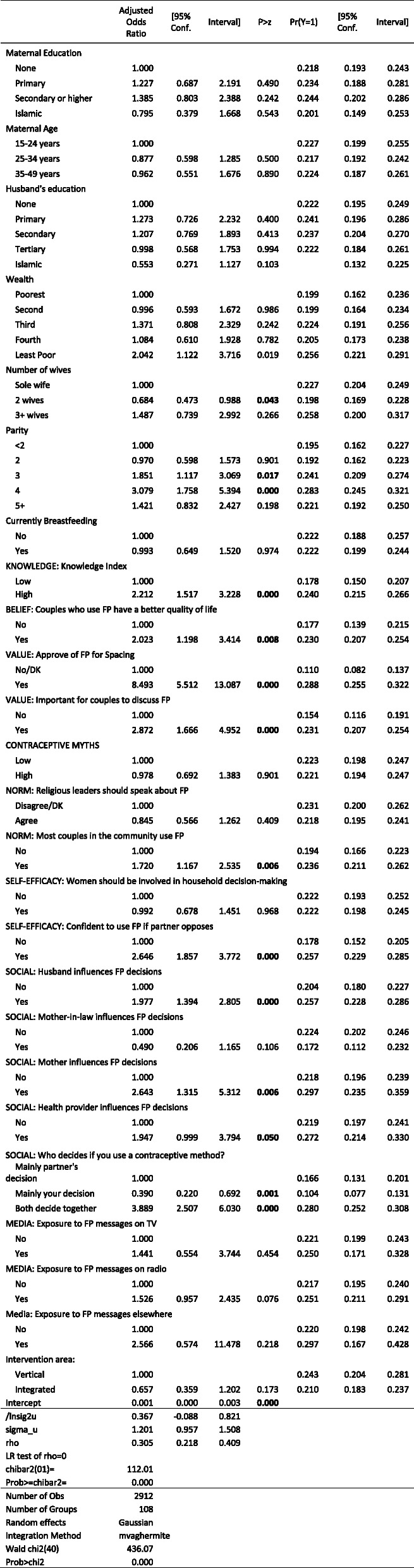
Mixed-Effects Logistic Regression: Ever spoken with husband about family planning

#### Approval of family planning

Because approval of family planning was strongly associated with modern contraceptive use, intentions to use, and discussions with husband, it is critical to understand the factors driving approval (Table 9). Unsurprisingly, greater knowledge and fewer contraceptive misconceptions were both associated with a greater likelihood of approval. Women above the median in contraceptive knowledge were five percentage points (51.6% versus 45.6%) more likely to approve of family planning. Women with contraceptive misconceptions were only 80% as likely as (43.0% versus 53.9%) to approve of family planning as women without such misconceptions. Other norms and values also mattered. Women who believed that it is important for couples to discuss family planning were 11.8 percentage points (51.8% versus 40.0%) more likely to approve of family planning, while women who believe that religious leaders should speak about family planning were 7.8 percentage points (52.3% versus 44.5%) more likely. This was the only effect of religious leaders in all of the analyses.

**Table 9:**
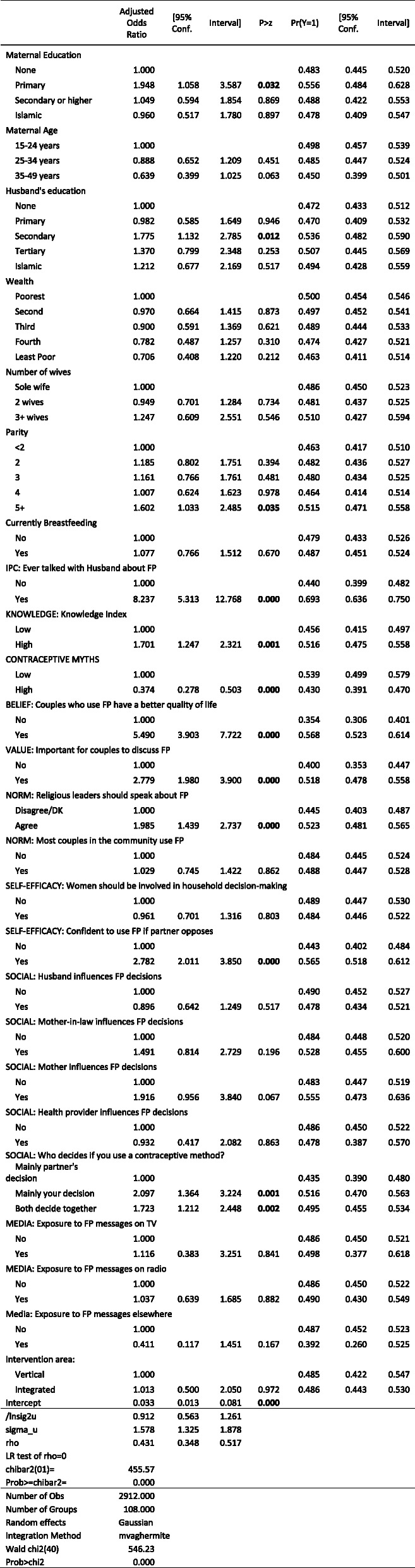
Mixed-Effects Logistic Regression: Approve of contraception for birth spacing

### Cross-cutting results

The influence of husbands appeared to be largely through family planning discussions and contraceptive decision-making. For example, women who had ever discussed family planning with their husbands were 14.7 percentage points (21.2% versus 6.5%) more likely to be currently using family planning, 16.6 percentage points (26.2% versus 9.6%) more likely to intend to use family planning, and 25.3 percentage points (69.3% versus 44.0%) more likely to approve of family planning as women who had never had such discussions (Tables 5, 6, 9 respectively).

Regression results further indicate that it is not simply the involvement of the husband that matters but rather that the husband needs to be involved in a joint decision-making process with the wife. Couples who make family planning choices together tended to have better family planning outcomes for all of the outcomes studied relative to couples in which unilateral decisions were made by the husband. For example, a woman who decides jointly with her husband about family planning was predicted to be 5.4 percentage points (14.5% versus 9.1%) more likely to be currently using modern contraception relative to a woman whose husband makes the decision himself – (Table 5). Similarly, predicted intentions to use family planning for a woman who made family planning decisions with her husband were 3.7 percentage points higher – 13.1% versus 9.4% - than for a woman whose husband decides unilaterally (Table 6). All outcomes, including discussions about children and family planning and approval of family planning, were higher for women who made joint decisions with their husbands relative to women whose husbands made family planning decisions unilaterally. Notably, women who have complete autonomy about family planning decisions are at least as likely to intend to use modern contraception in the next 6 months (16.9% relative to 13.1%) (Table 6) and to approve of family planning (51.6% versus 49.5%) (Table 9) as women who make joint family planning decisions.

We found little evidence that the position in which a woman is in the polygynous structure affected any of the outcomes (Tables 5-9). Specifically, controlling for other factors, the number of co-wives a woman has was not statistically related to current use of contraception, discussions about children and family planning nor approval of family planning. However, women in polygynous structures with three or more wives were 1.5 times more likely to intend to begin using family planning in the next 6 months, contrary to hypotheses related to rivalry amongst wives. Evaluation of this important motivator of fertility, however, was not the main focus of data collection.

We also found that, once ideational factors were controlled for, other variables, such as household wealth, women’s schooling, parity and husband’s education, were not significantly associated with these family planning outcomes.

#### Modeling impacts of changing ideational factors

To estimate what SBC programs can potentially achieve, we used the post-estimation marginal effects from the regression analysis results to simulate the magnitude of improvements in family planning outcomes that could be achieved in a world with improved ideation, that is, for example, if everyone had correct knowledge and held no contraceptive myths, or if everyone had positive beliefs surrounding family planning, or if everyone approved of family planning. We look at these impacts across the different domains of the ideational model – knowledge and risk perceptions, beliefs, values, norms, emotional (self-efficacy) and social influences.

**Fig. 1:**
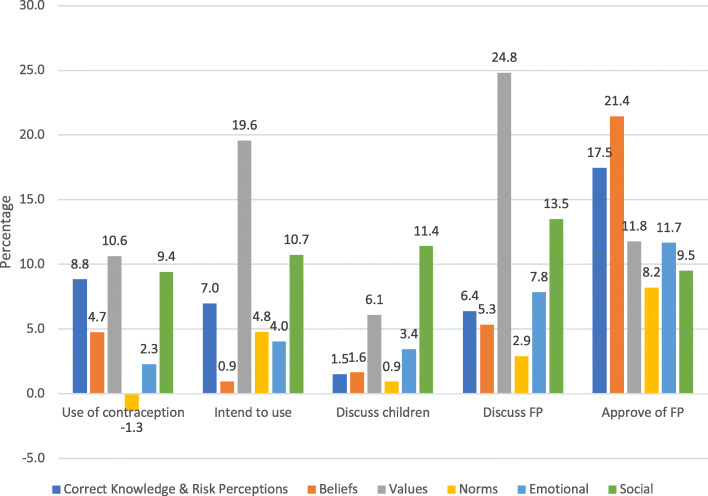
Marginal Effects from Ideational Factors

Values, specifically approval of family planning, appeared to have the largest impacts in general. For example, if all women approved of family planning for spacing, the estimated regression models indicate that contraceptive use could increase by 10.6 percentage points (from 13.4%), intentions to use contraception in the next six months could increase by 19.6 percentage points (from 14.7%), and the likelihood of discussing family planning with one’s husband could increase by 24.8 percentage points (from 22.5%) (Fig. 1). These are sizable impacts since they would result in a near doubling of contraceptive use and a more than 133% increase in contraceptive intentions, clearly desirable effects for SBC programs.

Achieving ideal knowledge and dispelling contraceptive myths amongst women could also have potentially large impacts, being associated with a greater likelihood of using contraception of 8.8 percentage points, a greater likelihood of intending to use contraception of 7.0 percentage points and increased approval of family planning of 17.5 percentage points. Social influences were far from negligible, influencing each outcome by 9 percentage points or more. Norms and beliefs tended to have the smallest impacts.

**Fig. 2:**
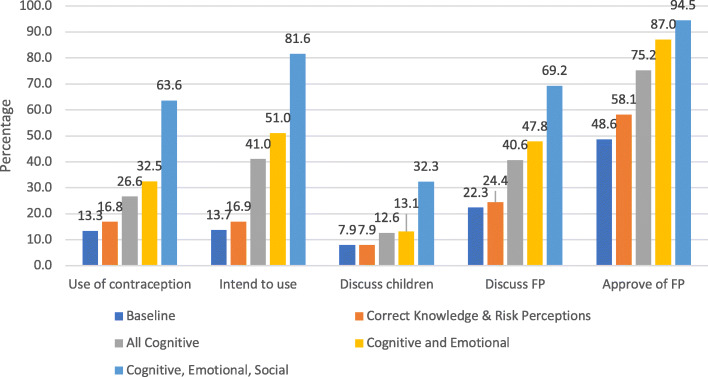
Improved Family Planning Outcomes from Improved Ideational Factors

The above simulations examine the individual influences on family planning outcomes from marginal changes in each of the ideational framework’s subdomains. We can also look at combinations of marginal effects, including what could be achieved as SBC programs achieved successive improvements in each of these subdomains. We examine the following scenarios: (1) if every woman had correct knowledge and held no contraceptive myths, (2) if every woman had ideal cognitive factors (e.g., high knowledge, positive beliefs, and values and norms supporting family planning), (3) if every woman had perfect cognitive and emotional factors (e.g., self-efficacy), and (4) if every woman had perfect cognitive, emotional and social factors (Fig. 2). These show how SBC programs could achieve impacts on contraceptive outcomes of many multiples. In a world of perfect ideation, for example, modern contraceptive use might reach as high as 63.6% of married women, intentions to use might reach 81.6% of non-users, discussions with husbands about the number of children to have and family planning might reach 32.3% and 69.2% respectively, and approval of family planning for spacing births could reach as high as 95% of women. Therefore, SBC programs that are able to shift these ideational factors may substantially improve a cascade of family planning outcomes.

## Discussion

Countries such as Nigeria are beset by long-standing patterns of high fertility, which can affect the health of mothers and their children. This study contributes to the evidence base for the design of family planning social and behavior change programs in high-fertility contexts in the following ways. First, it shows that commonly targeted family planning outcomes (e.g., modern contraceptive use, intentions to use modern contraception) are affected by ideational factors across a broad spectrum of cognitive, emotional and social domains. Several of these factors, such as improved knowledge of the benefits of contraception, increased approval of family planning and greater frequency of family planning discussions with husbands – are influential across many family planning outcomes. Approval of family planning represents an important barrier to use, and SBC programs that can overcome this barrier are likely to achieve important gains in contraceptive use. In this sample, we found that, even though only 43.2% of women approve of contraceptive use for spacing births, approval was associated with a nearly three-fold greater likelihood of contraceptive use and that approval itself could be significantly influenced by communications programs geared towards improving family planning knowledge and dispelling of contraceptive myths. Contraceptive knowledge was also a cross-cutting influencer. Knowledge worked not just through increased approval but also through its relationship with contraceptive discussions. Women above the median in contraceptive knowledge were 1.66 times more likely to have discussed fertility goals and 1.35 times more likely to have discussed family planning with husbands than women below the knowledge median. In turn, women who had discussed family planning with husbands were approximately three times more likely to be using modern contraception or to intend to use modern contraception.

Second, this work highlights that husbands are critical to family planning behaviors, even though family planning is often considered to be the woman’s domain. Couples in which family planning decisions are made jointly had better family planning outcomes across all outcomes studied relative to couples in which the husband is the sole decider. This is clearly an area where SBC programs can have impact, and SBC programs could maximize effectiveness by specifically engaging spouses in family planning promotion activities. Research elsewhere has noted the positive effects of male engagement [16, 33, 47]. As noted by one set of researchers, “the attitudes of men toward family planning can affect their partner’s contraceptive attitudes, even when spousal communication about reproductive health is not the norm” [16]. One shortcoming of this research is the absence of data from husbands regarding their knowledge, beliefs values and attitudes in order to inform SBC programs and family planning messaging for this key group.

Third, this work has provided support for the influence of other stakeholders in the family planning process. The influence of health providers, while cited by only a few women, was associated with a greater likelihood of using family planning, although the time order of involvement of health providers—*ex ante* before a woman made the decision to use family planning or as part of the decision about methods during a family planning visit at a health facility—is indeterminant. More research is needed on how best to engage health providers in family planning promotion activities and to identify key contact points that could increase their influence on birth spacing decisions. This would need to correspond with ongoing efforts to ensure sustainability of the quality of family planning services [48, 49].

Fourth, previous studies in Nigeria have evidenced how gender equitable attitudes and greater female autonomy are associated with a greater likelihood of contraceptive use [50, 51] while polygynous marital structures are associated with a lesser likelihood of use [51]. We have found limited evidence that polygynous family structures are associated with differences in family planning outcomes. We caution, however, that our data collection was not intended to measure the effects of polygynous family structures. Women were asked solely how many wives in total her husband had. We did not identify which wife – first, second, third – a woman was, nor how many children co-wives had, which could potentially bear upon the incentives that a woman faces when making choices about family planning use.

Fifth, many studies have stressed the need to support women and girls’ economic and social empowerment, largely through increasing the school enrollment of girls, which could both improve women’s health literacy and strengthen employment prospects for girls and women. We were unable to detect strong differences in outcomes by education levels. However, it is likely that such differences were already accounted for in the ideational factors. Women with a secondary or higher level of education were 31 percentage points (86.6% versus 55.4%) more likely to believe that couples who use family planning have a better quality of life, 26 percentage points (90.8% versus 64.8%) more likely to agree that it is important for couples to discuss contraception, and 35.3 percentage points (78.9% versus 43.6%) more likely to be above the median in contraceptive knowledge relative to women with no education. Through these ideational factors, education can serve as an important conduit for making better reproductive health choices.

Finally, we were unable to identify a strong direct influence of religious leaders on family planning decisions, although admittedly our data did not permit detailed analyses of such influences. No women reported that religious leaders influence their contraceptive use decisions, and only half of women believe that religious leaders should speak publicly about family planning. That belief was not associated with any of the family planning outcomes under study with the exception of contraceptive approval. Women who agreed with that statement were 18% more likely to approve of family planning. The influence of religious leaders may therefore logically flow through the values that women hold regarding family planning. Previous studies have highlighted the importance of religious leaders and have noted that exposure to family messages from religious leaders was positively associated with contraceptive use [52]. Previous researchers have also emphasized “the need to empower religious leaders to be advocates for family planning and to emphasize the positive position of Islamic religious tenets on contraception through multiple channels” [20]. Subsequent work will evaluate more fully the impact of religious leaders on contraceptive outcomes.

This study faced several important limitations. First, no information was available on the supply side of the contraceptive use decision, which necessitates the assumption that supply-side factors (e.g., prices, access, quality) are not correlated with other covariates in the models, e.g., that family planning norms, attitudes and values do not differ across different supply environments. To the extent that such ideational factors tend to be better in areas with higher quality family planning services, which would likely directly affect contraceptive uptake, our estimates of the effects of these ideational factors may be over-stated. Many studies have incorporated supply-side characteristics into demand analyses for family planning [53–56], and future work will hopefully add this dimension to the northwestern Nigerian context.

Second, this study has not used a sample of all women of reproductive age but rather a sample of women who had a completed pregnancy in the last 2 y. This sample therefore may have different views and experiences of family planning than a larger sample of women of reproductive age and hence the estimated relationships may not be reflective of all such women.

Third, this study has identified associations and not necessarily causal influences. This is a limitation faced by much of the ideational literature and is largely tied both to the inability of researchers to control exposure and to the cross-sectional nature of the data collection, which relies upon retrospective recall of events in which the time order of influencers and behaviors is unclear. The ideational theory posits causal relationships but what has been established here, and in nearly all similar studies with a few exceptions [57], are correlations. Using data that reflect a snapshot in time with retrospective information, it is virtually impossible to establish the time-order of events (e.g., when was knowledge attained – before, during or after uptake of modern contraception?) and to eliminate issues of reverse causality. Many ideational factors, for example, may actually be self-determined through the process of using family planning. Through use, individuals may gain greater knowledge, develop greater self-efficacy to use contraception, develop more accurate perceptions of risk, become more likely to discuss family planning with husbands, and develop values such as believing that couples who use family planning have a better quality of life. Many possibilities exist to explain the associations.

In this paper, we have spent a good bit of time examining the influence of discussions between husbands and wives on family planning outcomes. Ideally, SBC programs would provide health information to couples, the couples would discuss fertility goals, and then they would make an informed choice to adopt a contraceptive method or not. But our reference period is whether or not a woman has ever had a discussion with her husband about family planning. We do not know for certain if the discussion preceded contraceptive use or if it occurred subsequently. In the former case, causality could perhaps be inferred. Discussions resulted in uptake of family planning. In the latter case, causality would not be present; discussions with husbands and contraceptive uptake would simply be co-occurring events. Hence, it is impossible to distinguish between the *ex-ante* influences of these variables from the *ex-post* changes that arise from the process of using modern contraception. Assuming that these ideational factors represent unidirectional causal influences may overstate their effects on family planning outcomes.

We recommend that future researchers more fully explore both experimental designs to control for unobservable factors that may simultaneously influence ideational factors and the outcomes they are hypothesized to affect. We also recommend panel data collection, which may better tease out the time-order of events. Ideational factors, measured at one wave of data collection, could then be linked to changes in family planning outcomes – use of modern contraception and intentions to use – in subsequent waves, lending greater credence to causal pathways [57].

## Conclusion

High fertility and low contraceptive use in northwestern Nigeria are influenced by numerous factors, including social norms for high fertility, pro-natal cultural and religious beliefs, misconceptions about contraceptive methods, and gender inequalities. This study has shown that better family planning outcomes are associated with a variety of theorized drivers of family planning behaviors, including personal approval of modern contraception, communication with spouse/partner, correct knowledge of contraceptive benefits, accurate risk perceptions, and self-efficacy to use contraception. The implication is that well-designed social and behavior change programs that target these potential drivers can have large potential benefits. Our analysis showed that improving contraceptive knowledge and risk perceptions alone could increase modern contraceptive use by approximately 8.8 percentage points and approval of modern contraception by 17.5 percentage points. The latter effect would propel further improvements in contraceptive use. Women, however, do not make family planning decisions in a vacuum, and this analysis has further shown the important effects of social influences from husbands, family members, and health care providers. To bolster the effects of SBC messaging on women’s behaviors, SBC programs would do well to target those latter groups in addition to targeting the women users themselves.

## Data Availability

The data for this study, the 2019 Breakthrough Research Behavioral Sentinel Surveillance Survey, are publicly available in a deidentified format through USAID’s Development Data Library (DDL) at data.usaid.gov.

## Abbreviations

B-A/N: Breakthrough ACTION/Nigeria
EA: Enumeration Area
FP: Family Planning
IUD: Intrauterine Device
LAM: Lactational amenorrhea method
LGA: Local Government Area
NPC: National Population Commission
NDHS: Nigeria Demographic and Health Survey
Obs: Observations
SBC: Social and Behavior Change
USAID: United States Agency for International Development

## Footnotes

1 The complete list of myths included: “use of some contraceptives can make a woman permanently infertile,” “contraceptives can harm a woman’s womb,” “contraceptives can reduce a man’s sexual urge,” “contraceptives can reduce a woman’s sexual urge,” “contraceptives can cause cancer,” “contraceptives can give you deformed babies,” “women who use contraception end up with health problems,” and “women who use contraceptives may become promiscuous.”
